# Machine learning models for efficient characterization of Schottky barrier photodiode internal parameters

**DOI:** 10.1038/s41598-023-41111-7

**Published:** 2023-08-26

**Authors:** Richard O. Ocaya, Andronicus A. Akinyelu, Abdullah G. Al-Sehemi, Ayşegul Dere, Ahmed A. Al-Ghamdi, Fahrettin Yakuphanoğlu

**Affiliations:** 1 Department of Physics, University of the Free State, P. Bag X13, Phuthaditjhaba, 9866 South Africa; 2 Department of Computer Science and Informatics, University of the Free State, P. Bag X13, Phuthaditjhaba, 9866 South Africa; 3 Department of Chemistry, Faculty of Science, King Khalid University, P.O. Box 9004, Abha, 61413 Saudi Arabia; 4 Research Center for Advanced Materials Science, King Khalid University, P.O. Box 9004, Abha, 61413 Saudi Arabia; 5 Unit of Science and Technology, Faculty of Science, King Khalid University, P.O. Box 9004, Abha, 61413 Saudi Arabia; 6 Vocational School of Technical Science, Department of Electric and Energy, Firat University, Elazig, Turkey; 7 Department of Physics, Faculty of Science, King Abdulaziz University, Jeddah, 21589 Saudi Arabia; 8 Department of Physics, Faculty of Science, Firat University, Elazig, Turkey

**Keywords:** Materials science, Photonic devices, Physics, Characterization and analytical techniques, Computer science, Silicon photonics, Information technology, Computational science

## Abstract

We propose ANN-based models to analyze and extract the internal parameters of a Schottky photodiode (SPD) without presenting them with any knowledge of the highly nonlinear thermionic emission (TE) expression of the device current. We train, evaluate and demonstrate the ML models on thirty-six private datasets from three previously published devices, which denote current responses under illumination and ambient temperature of graphene oxide (GO) doped p-Si Schottky barrier diodes (SBDs). The GO doping levels are 0%, 1%, 3%, 5%, and 10%. The illumination ranged from dark (0 mW/cm^2^) to 30 mW/cm^2^. The predictions are then made completely at the intensity of 60 mW/cm^2^. For each diode, some values of the barrier height ($\phi$), ideality factor (*n*), and series resistance ($R_s$) independently calculated using the Cheung–Cheung method were included in the training dataset. The predictions are done at unspecified intensities on the model development data at 80 and 100 mW/cm^2^, and on external data at 5% and 20% GO doping which were not part of the development dataset. The ANN achieved a mean square error and mean absolute error score below 0.003 across all datasets. This demonstrates the effective learning capabilities of the ANN models in accurately capturing the photo responses of the photodiodes and accurately predicting the internal parameters of the Schottky Barrier Diodes (SBDs), all without relying on an inherent understanding of the thermionic emission (TE) equation for SBDs. The ANN models achieved high accuracy in this process. The proposed ML models can significantly reduce analysis time in device development cycles and can be applied to other datasets in various fields.

## Introduction

Today, artificial intelligence (AI) systems are demonstrating abilities that match or surpass skilled human performance in many fields, a feat that was barely possible 1 year ago and that is evolving at an unprecedented rate^1^. There is a growing focus on applying AI techniques to data extraction and analysis in the physical and applied sciences^2^. Only a few studies have applied ML-based algorithms to model the internal parameters of photodiodes. Ruiz Euler et al.^3^ utilized deep neural networks (DNN) to optimize multi-terminal nanoelectronics devices. They employed the gradient descent algorithm^4^ and achieved successful predictions of device functionality in disordered networks of dopant atoms in silicon. El-Mahalawy and El-Safty^5^ employed the Quantum Neural Network (QNN) to model the characteristics of the NTCDA/p-Si UV photodiode, accurately capturing trends and extrapolating unknown current values under different illuminations. ML algorithms have also found applications in laser welding^6–8^, optical photodiodes^9,10^, organic diodes^11^, and photonics^12^.

## Theoretical background

**Figure 1:**
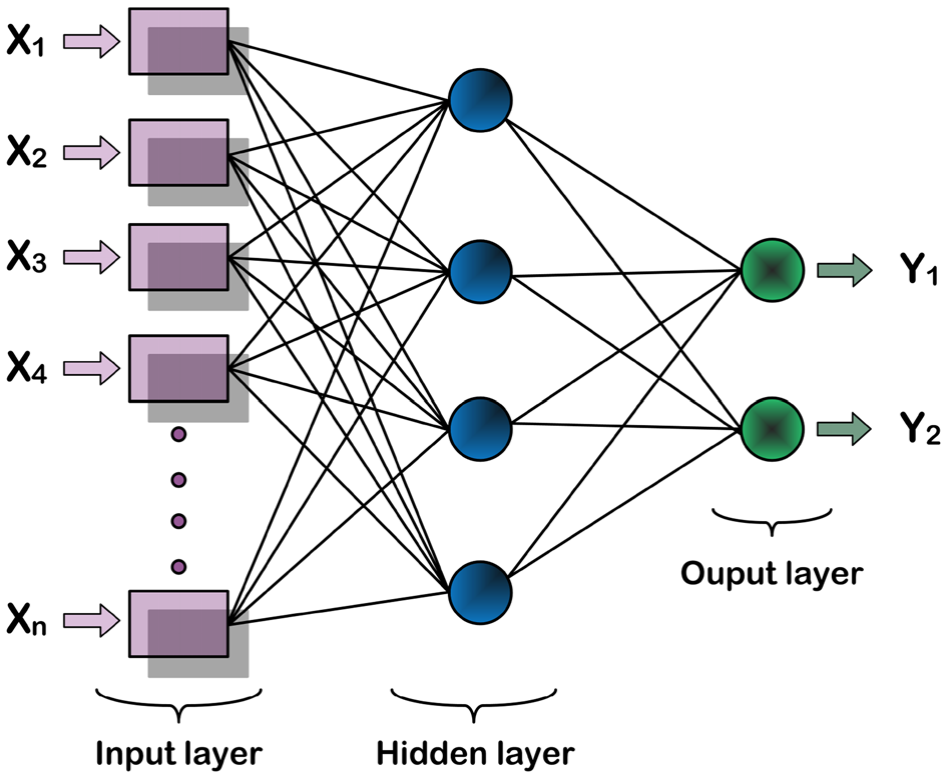
A schematic depiction of a typical ANN model.

In this study, we assemble, train, and apply ML to evaluate the internal parameters of semiconductor photodiodes (SPDs) when their current responses to illuminations are empirically known. This is a standard experiment for semiconductor diodes. The current response of an SPD is governed by the TE equation. This is a complex equation that depends on the aforementioned internal parameters $\phi$, *n*, $R_s$, on the applied voltage bias *V*, and on the ambient parameters i.e. the absolute device temperature *T*, and illumination, *P*. An empirical data point in a typical SPD measurement (at a given *P* and *T*) consists of the external, observable diode current *I*, and *V*. Incidentally, in the TE model, *I* is circularly dependent on itself in combination with $R_s$, *V*, *T*, $\phi$, and *n* according to the expression

1 $$\begin{aligned} I=AA^*T^2\exp \Big (-\frac{q\phi }{kT}\Big )\exp \Big (\frac{qV-IR_s}{nkT}\Big )\Big \{1-\exp \Big (-\frac{qV-IR_s}{kT}\Big )\Big \}, \end{aligned}$$

where *q* is electronic charge, *k* is the Boltzmann constant, *A* is the diode area, $A^*$ is the Richardson constant^13–16^. For a given SPD, the interest is to characterize *n*, $R_s$, and $\phi$. Evidently, Eq. (1) is extremely difficult to evaluate for these parameters, with many methods having been devised over the last five decades. Many are still in use, but almost all rely on heavy simplifying approximations owing to the typically non-zero $R_s$ in real devices^17–20^. One such method is the Cheung–Cheung method which was developed in the 1980s^18^. It relies on two functions that are linear in the current:

2 $$\begin{aligned} \frac{dV}{d\ln I}= & {} R_sI+\frac{nkT}{q},\nonumber \\ H(I)= & {} R_sI+n\phi = V-\frac{nkT}{q}\ln \Big (\frac{I}{AA^*T^2}\Big ), \end{aligned}$$

with the symbols as previously defined. The method gives two estimates of $R_s$. The intercepts from the first and second plots then lead to an estimate of *n* and $\phi$, respectively. For the datasets used in this study, $A^*$=32 A/K$^2$cm$^2$, *A*=1mm, and *T*=300K. Then, we assemble, train, and apply an ANN machine language model to the empirical datasets to evaluate the internal parameters of a p-Si/Au SPD without explicitly presenting Eq. (1) to the models. For background, we briefly shall describe the ANN model, but the detailed operational principles can be found in several sources. (Fig. 1)

In brief, the ANN model simulates interconnected neuron networks (through interconnected nodes) that are inspired by the human brain. The depiction shows an input layer, one or more hidden layers that extract input and output features and patterns, and an output layer that produces the final classification. The *j*-th output, $Y_j$, is computed using weights $w_i$ on inputs $X_i$ according to

3 $$\begin{aligned} Y_j=\sum _{i=1}^nw_iX_i+b. \end{aligned}$$

A bias factor, *b*, is included for flexibility in training the model. In this study, the weighted sum is input to the efficient, nonlinearity-inducing mathematical function called the rectified linear unit (ReLU) to determine whether or not a node with critical inputs is activated. The function ReLU is defined as

4 $$\begin{aligned} f(x)=\max (0,x)= {\left\{ \begin{array}{ll} x &{} \text {if } x>0\\ 0 &{} \text {otherwise}, \end{array}\right. } \end{aligned}$$

where *x* refers to the input to a neuron in the network.

In this study, we trained and evaluated the ANN ML model on thirty-six private experimental datasets from three different SBDs, denoted D1, D2, and D3, using Google Colaboratory^21^. The dataset is derived from our previously published SPDs^22–24^. They contain responses to the illumination falling on three photodiodes doped with 0%, 1%, 3%, 5%, and 10% graphene on the p-type silicon substrate material. Each SPD is subjected to dark up to 30 mW/cm^2^ illumination. The datasets also contain the calculated values of the following parameters for each diode: barrier height ($\phi$), ideality factor (*n*), and the series resistance ($R_s$). The results demonstrate that the ANN achieved a mean square error (MSE) and a mean absolute error (MAE) score of less than 0.003 for all datasets. Thus, the ANN ML model efficiently learned the photo responses of the photodiodes and correctly predicted their barrier height, ideality factor, and series resistance with extreme accuracy. We show that the ML model does not need prior knowledge of the mathematical TE model in any form to reach its predictions. We argue that ML modeling should be seen as a complement to researchers by adding a new, rapidly evolving tool into their arsenal. These tools can substantially reduce the time for analysis in the device development cycle and can be adapted to other areas and fields.

## Results

### The ANN model

Table 1 shows the train and validation accuracy for the ANN model after 30 epochs. An Epoch marks the processing of all data once. It typically involves several iterations, which can involve data batches of a specified size.

**Table 1:** Averaged MSE and MAE performance of the ANN models for three different SBDs, D1, D2, and D3.

Illumination (mW/cm^2^)	GO:CoPc (= D1)			GO:PCBM (= D2)			GO:Coumarin (=D3)		
	Doping	MSE	MAE	Doping	MSE	MAE	Doping	MSE	MAE
	%	(10^-3^)	(10^-3^)	%	(10^-3^)	(10^-3^)	%	(10^-3^)	(10^-3^)
0	0	2.33	9.22	0.5	1.49	2.52	3	1.25	1.64
10	0	1.93	3.34	0.5	1.55	2.74	3	1.28	1.43
30	0	1.44	7.66	0.5	1.93	2.51	3	1.43	4.00
60	0	1.59	5.60	0.5	1.19	1.82	3	1.23	2.60
0	1	1.22	2.57	3	1.11	1.37	5	1.47	1.48
10	1	1.94	2.91	3	1.14	2.09	5	0.91	0.80
30	1	1.54	6.75	3	2.56	3.82	5	1.89	1.91
60	1	1.78	2.05	3	1.71	1.35	5	1.48	1.25
0	10	1.35	4.10	10	1.34	2.99	10	1.35	0.42
10	10	1.78	3.29	10	1.18	1.70	10	0.76	0.69
30	10	1.88	4.39	10	1.42	2.33	10	1.08	0.94
60	10	2.24	3.89	10	1.81	3.25	10	1.23	1.52

**Figure 2:**
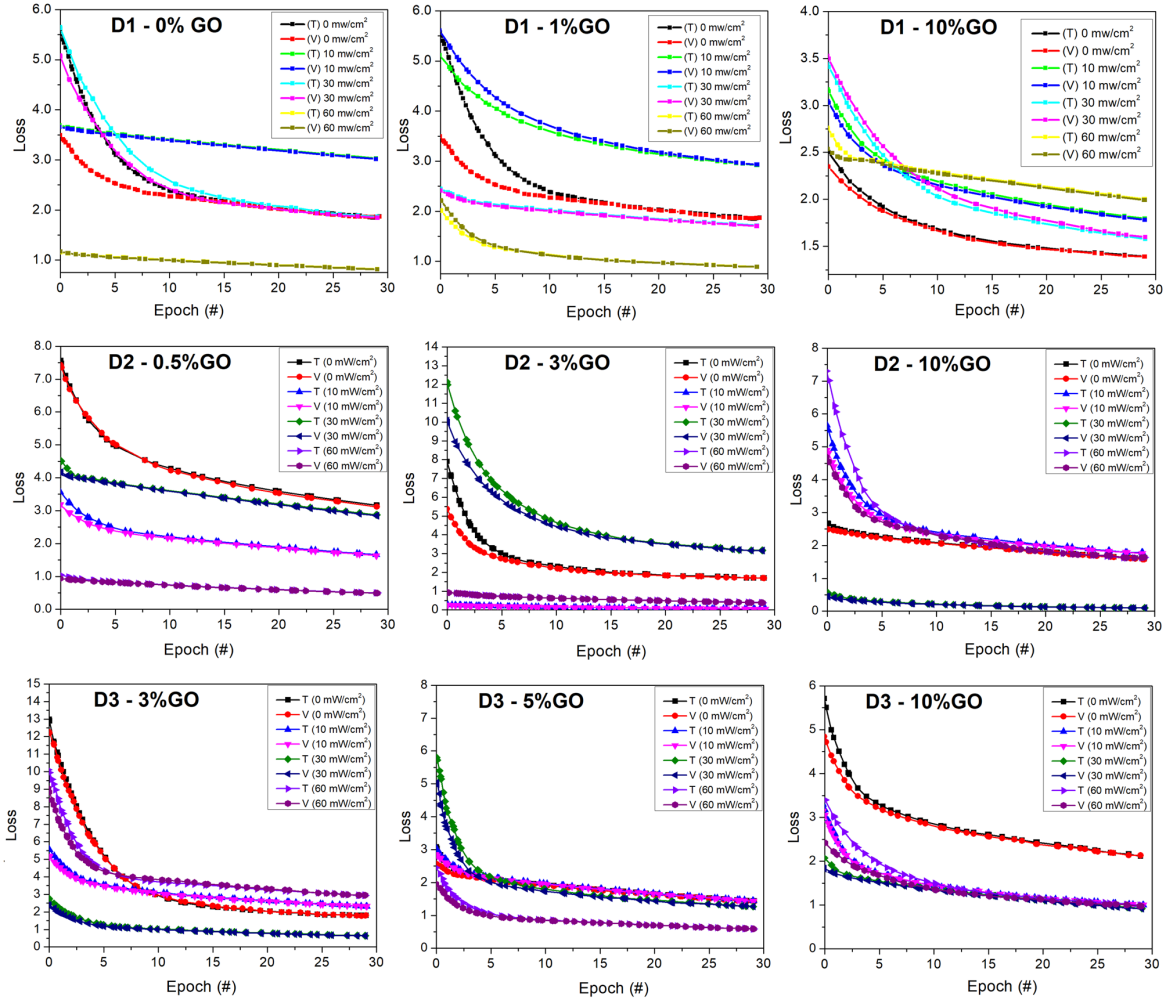
Collated plots showing the training (curves T) and validation (curves V) losses for the ANN ML model applied to three SBDs D1, D2, and D3. All the plots have a scale factor of 10^−3^. The doping levels range from 0 to 10% GO, and the illuminations from 0 to 60 mW/cm^2^. The original simulation images are available^21^.

Additionally, the table provides the average test accuracy for each model, which was calculated by summing the three test accuracies and dividing them by three. These findings suggest that the ANN model achieved good accuracy in predicting the target variables. The results also indicate that the model’s performance varied depending on the dataset used for training. This could be due to differences in the characteristics of the datasets, such as the range and distribution of the variables. Further investigations are needed to determine the factors affecting the model’s performance and to optimize its training parameters. In summary, the ANN model trained on thirty-six datasets from three devices using two independent and three target variables showed promising results in predicting $\phi$, *n*, and $R_s$. Figure 2 shows the training loss and accuracy curve for some models. The plots show the model’s performance for 30 epochs. The figures show that both the train—validation accuracy curve distance, and the train—validation loss curve distance is small, indicating that there is no overfitting in the ANN models.

**Figure 3:**
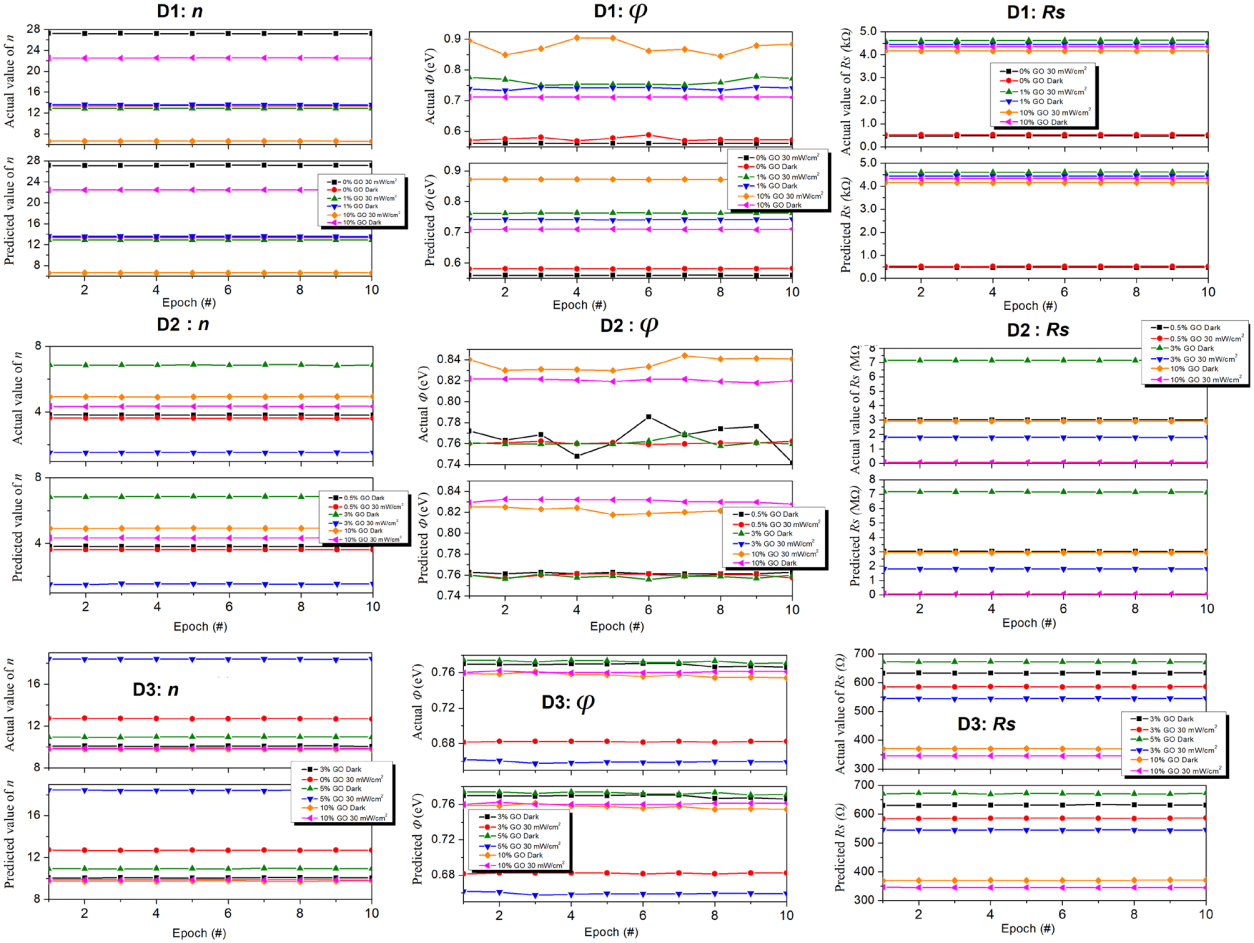
Plots showing comparisons between the actual and the ANN model predicted values of *n*, $\Phi$, and $R_s$ for three SBDs, D1, D2, and D3. For reasons of limited space, only values at dark and 30 mW/cm$^2$ intensities are compared^21^.

The comparison is depicted in Fig. 3, where space limitations allowed us to present the comparison for ten predicted and actual values per dataset. The plots reveal a negligible difference between the predicted and actual values. This compellingly demonstrates the accuracy of the ANN models’ predictions, as the predicted values align satisfactorily with the expected values.

Furthermore, it affirms the effective learning of the photodiodes’ light responses by the ANN models, leading to accurate predictions of barrier height, ideality factor, and series resistance. The performance of the ANN models exhibited variability based on the training dataset utilized. This variability may arise from disparities in dataset characteristics, encompassing variables’ range and distribution. To comprehensively understand the factors influencing the model’s performance and optimize training parameters, further investigations are warranted. In summary, the ANN model trained on twelve datasets, incorporating two independent variables and three target variables, demonstrated promising outcomes in accurately predicting $\Phi$, *n*, and $R_s$.

## Discussion

The aforementioned machine learning (ML) languages have demonstrated the ability to deduce patterns in data pertaining to the transfer characteristics of the SPD without any prior knowledge of the device physics or the TE equation. This is achieved through training on a small set of data, allowing the ML model to generalize and make accurate predictions on unseen data. The current-voltage relationship of SPDs is governed by the highly non-linear TE equation. In the context of parameter extraction for a given SPD, it is essential to linearize specific regions of the *V*-*I* characteristic. Many methods, like the previously described Cheung–Cheung method, are viable to accomplish this task but require a carefully selected voltage range to give accurate results. As a consequence, different individuals may select different ranges, leading to a significant variance in the extracted parameters even for a given SPD. During the implementation of the ANN models, it was observed that the algorithms rapidly and unexpectedly converged to the optimal ranges of applied bias across all instances. The study’s findings demonstrate the effectiveness of using ANN ML-based algorithms to accurately model the light responses of photodiodes. Through extensive training and evaluation of data collected from three photodiodes, the models successfully predicted critical parameters, such as barrier height, series resistance, and ideality factor. Importantly, the models also exhibited the ability to estimate photodiode light responses under varying illuminations and voltage settings, demonstrating their broad applicability. Furthermore, the models proved capable of predicting responses with minimal error, from 0 to 100 mW/cm^2^. However, complete reliance on machine learning (ML) models may not provide a comprehensive understanding of peculiarities in the data, such as negative differential conductance regions or breakdowns. Moreover, this study only explored one type of Schottky barrier diode (SBD) with p-Si/Au construction. However, SBDs with different constructions exhibit similar characteristics to the present devices, and it is highly likely that the same models can be used to determine their internal parameters. Further investigations are necessary to verify this, as the scope of this work is not intended to be exhaustive, but rather to demonstrate the potential of ML tools. Researchers can utilize these models to minimize the time and resources required for conducting experiments. These findings provide an exciting avenue for future research through the application of ML techniques to model complex and highly nonlinear systems, thereby enhancing our overall understanding of their behaviors. Finally, the dataset^25^ and models^26–28^ are available upon request to enable independent evaluation.

## Methods

### Dataset creation

The datasets presented to the developed ML models are based on *V* and *I* data points measured on three different published SBDs: D1 = Al/GO:CoPc/p-Si/Au^22^, D2 = Al/pSi/GO:PCBM/Au^23^, and D3 = Au/GO:Coumarin/p-Si/Al^24^. The publications summarize the instantaneous estimates of $\phi$, *n*, and $R_s$ using the Cheung–Cheung functions in Eq. (2). Table 2 shows the known results of the Cheung–Cheung method for all three diodes with 0%, 1%, 3%, 5%, and 10% GO content.

**Table 2:** Empirical results using the Cheung–Cheung functions for SBDs D1, D2, and D3. The entries at 80 and 100 mW/cm^2^ are the predictions by the ANN model.

SBD	Dataset	*n*	$R_s$ (k$\Omega$)	$\phi$ (eV)	*n*	$R_s$ (k$\Omega$)	$\phi$ (eV)	*n*	$R_s$ (k$\Omega$)	$\phi$ (eV)
	(mW/cm^2^)	0%GO			1%GO			10%GO		
D1	0	13.28	0.516	0.583	13.55	4.42	0.737	22.44	4.32	0.707
	10	23.78	0.509	0.579	12.44	4.90	0.764	20.91	4.49	0.686
	30	27.15	0.468	0.563	12.85	4.60	0.757	6.60	4.13	0.874
	60	23.00	0.516	0.577	9.89	6.60	0.807	10.86	2.07	0.758
	80	23.85	0.497	0.572	12.11	4.90	0.764	11.16	1.58	0.753
	100	22.23	0.502	0.576	11.00	4.54	0.776	12.39	1.72	0.736
		0.5%GO			3%GO			10%GO		
D2	0	3.80	3022	0.761	6.84	7128	0.762	4.89	2934	0.819
	10	3.57	19.56	0.764	1.02	2860	0.996	4.07	1017	0.849
	30	3.62	18.58	0.761	1.53	1803	0.944	4.33	678	0.835
	60	3.91	17.37	0.753	1.62	1613	0.935	4.50	481	0.827
	80	4.07	15.95	0.748	4.01	848	0.817	4.78	311	0.815
	100	5.91	13.41	0.721	4.25	737	0.813	5.31	232	0.801
		3%GO			5%GO			10%GO		
D3	0	10.03	0.632	0.768	10.93	0.670	0.772	9.75	0.369	0.760
	10	14.79	0.393	0.675	12.57	0.743	0.729	10.25	0.313	0.751
	30	12.65	0.582	0.696	18.33	0.543	0.658	9.86	0.345	0.756
	60	14.20	0.472	0.680	20.74	0.448	0.637	9.42	0.370	0.759
	80	12.60	0.597	0.712	15.3	0.662	0.685	9.95	0.336	0.752
	100	19.23	0.287	0.633	18.91	0.476	0.650	9.90	0.381	0.739

**Figure 4:**
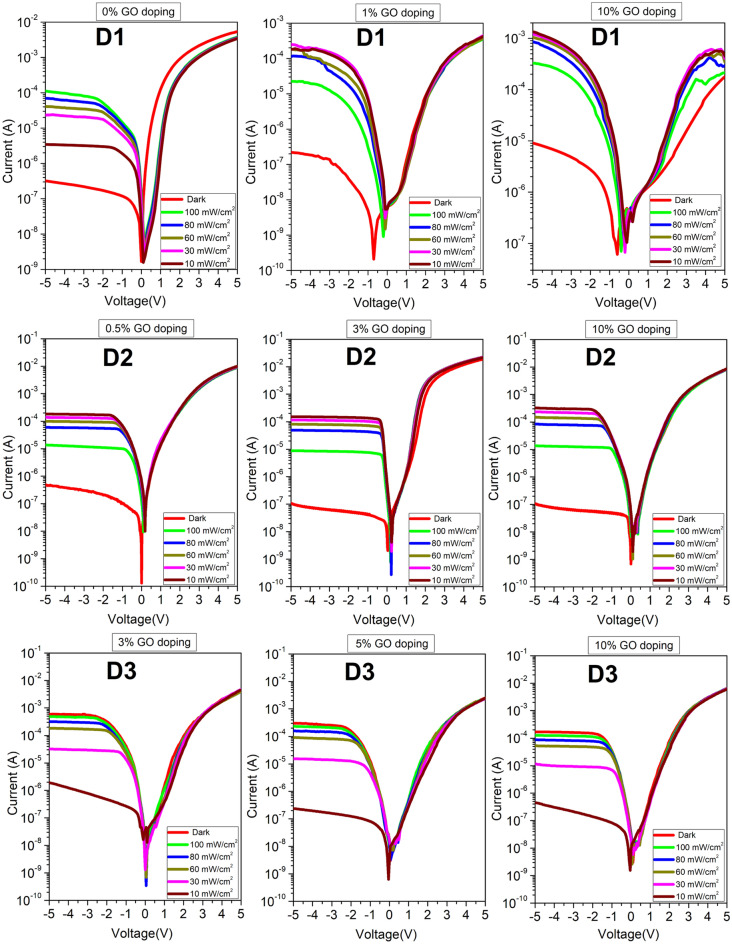
The plots of the measured current-voltage characteristics for the three diodes D1, D2, and D3 with various dopants and doping concentrations. Each set of I-V characteristics is measured over the 0 to 100 mW/cm^2^ illumination intensity range.

Each dataset entry therefore has the structure (*V*, *I*, $\phi$, *n*, $R_s$) for each illumination intensity. The four illumination intensities used are 0 mW/cm^2^ (dark), 10, 30, and 60, all measured in mW/cm^2^. Thirty-six private datasets denoting 0%, 1%, 3%, 5%, and 10% doping levels were collected from three different devices. Twelve of the datasets were collected from D1 with 101 measured (I, V) samples, twelve from D2 with 201 samples, and twelve from D3 with 251 samples. Figure 4 shows the plot of the raw empirical data collected for these diodes over the illumination range from dark to 100 mW/cm^2^. Only the 0–60 mW/cm^2^ intensities were used in the development of the models. The 80 and 100 mW/cm^2^ intensities were used as data for prediction during the ML model development.

In all, there are 36 datasets consisting of 101, 201, and 251 sample points, respectively. They represent actual measurements along the current-voltage characteristics, from − 5 V to + 5V, including 0, in steps of 0.1, 0.04 V, and 0.05 V, respectively.

The datasets were standardized before training and contain the calculated $\phi$, *n*, and $R_s$ for each diode. Standardization was done to ensure that all the responses in the dataset contribute equally to the applied ML model.

### The overall approach

**Figure 5:**
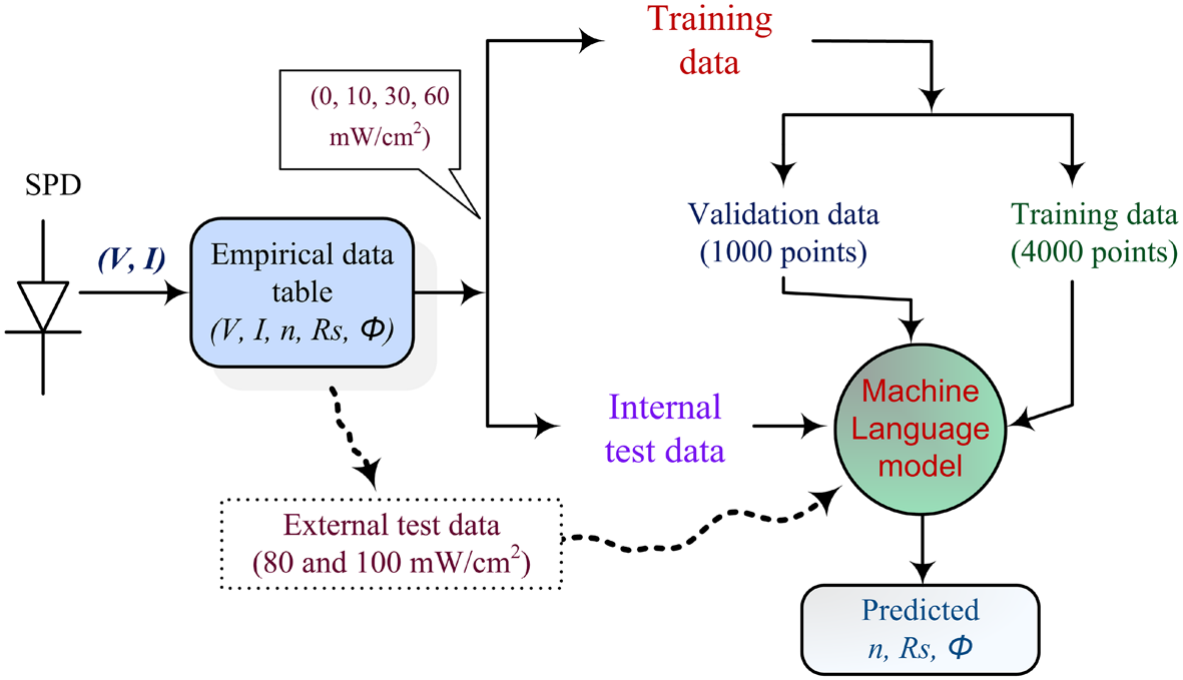
A block schematic representation of the machine language models and the data flows in the processing.

**Figure 6:**
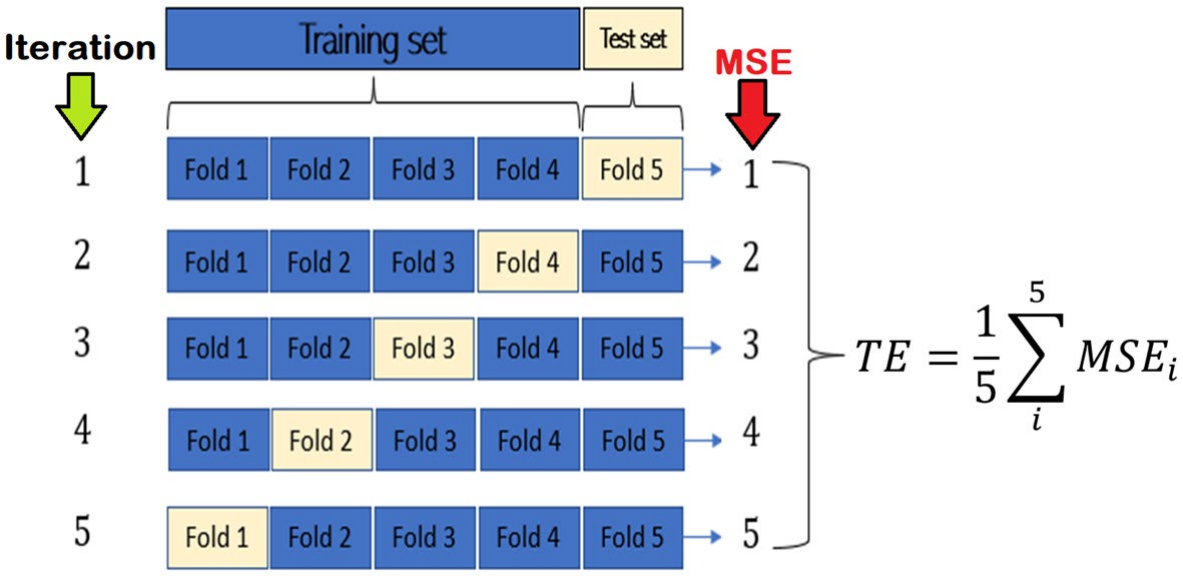
The five-fold cross-validation procedure. TE is the total error.

We utilize ANN models to assess internal parameters of a p-Si/Au SPD using empirical datasets, without explicitly presenting Eq. (1). The ANN model comprises an input layer and an output layer, with a specific number of layers determined through experimentation. The input layer consists of two neurons, while the output layer consists of three neurons representing $\Phi$, *n*, and $R_s$. The ANN architecture and training parameters are depicted in Fig. 1 and Table 3, respectively. Figure 5 shows the data flow in the ANN ML used in this work. To accommodate the dataset size, we employed five-fold cross-validation to evaluate the ANN models, ensuring a more reliable estimation of performance on unseen samples. The five-fold cross-validation procedure utilized the KFold cross-validation function from the scikit-learn ML library^29^. Figure 6 illustrates the steps involved in the five-fold cross-validation.

**Table 3:** Training parameters for the ANN used in this work.

Dataset	Values
Batch size	32
Number of Epochs	30
Learning rate	0.001
Max Num value	2
L2 regularization factor	0.001
Optimizer	Adam^30^

The dataset is split into five-folds, and the experiment is executed over five iterations. In each iteration, four folds are utilized for training the ANN model, while the remaining fold is used for testing. This process is repeated until all five folds have been employed for training and testing. Throughout each iteration, the MSE and the MAE are calculated and recorded. Ultimately, the average MSE and MAE are computed at the conclusion of the final iteration.

### Training and model testing

The ANN model was first trained and then cross-validated by the five-fold approach in Fig. 6. The training and validation made use of thirty-six (3x12) different datasets, based on the parameters in Table 3. The 3 $\times$ 12 datasets comprised 101, 201, and 251 (I, V) samples, respectively.

**Figure 7:**
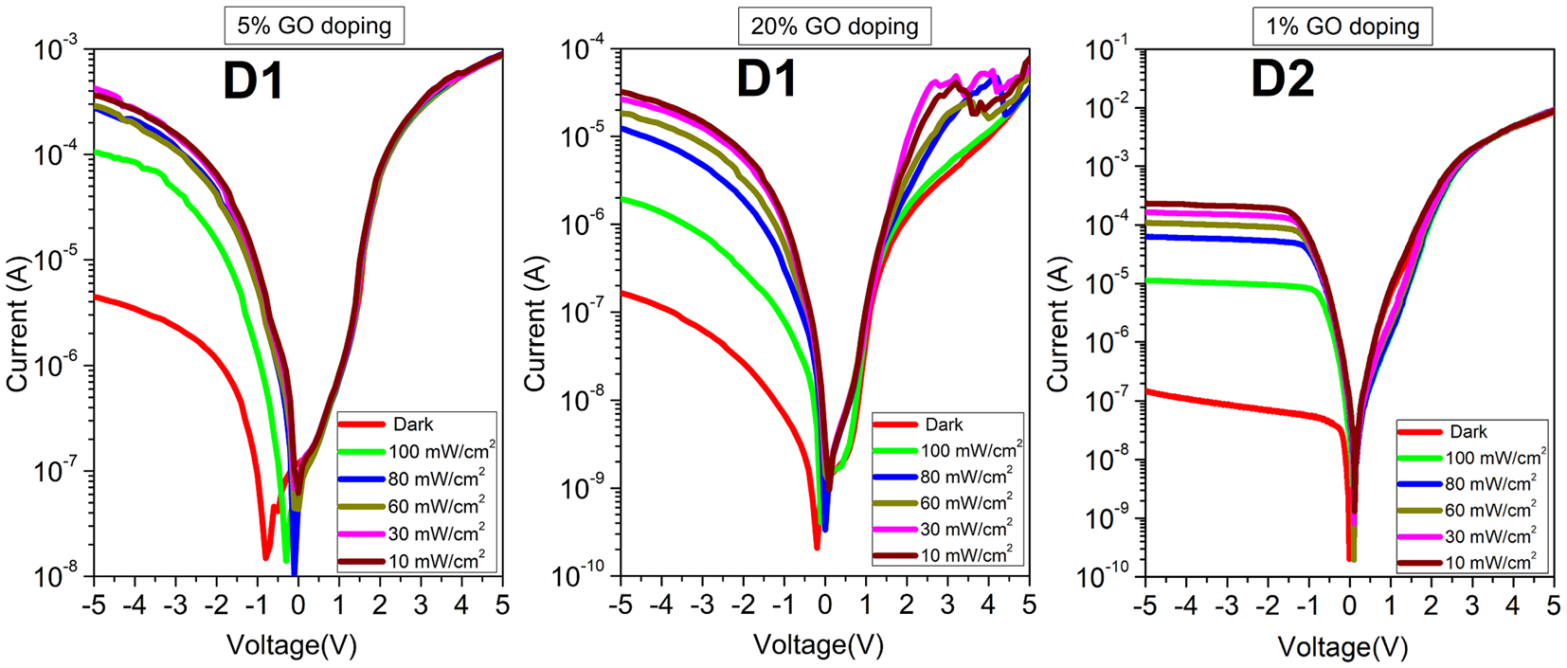
Plots of additional original datasets for D1 at 5% and 20% GO doping levels, and D2 at 1% GO. These extra data were not used in the ML model development stages but for further validation.

Finally, the trained model was tested on all samples from the original datasets. Table 1 also shows the MSE and MAE for the developed ANN model for D1, D2, and D3. Figure 7 plots the current-voltage characteristics for external data that were used to validate the ML models further.

### Performance metrics

The performance of the ANN models was assessed by the aforementioned metrics: MSE and MAE. A low MSE and MAE indicate a highly accurate model, with a zero MSE signifying a perfect match between predicted and actual values. Mathematically,

5 $$\begin{aligned} \text {MSE} = \frac{1}{N}\sum _{i=1}^N(V_{a,i} - V_{p,i})^2\quad \text {and}\quad \text {MAE} = \frac{1}{N}\sum _{i=1}^N|V_{a,i} - V_{p,i}|, \end{aligned}$$

where *N* is the number of samples in the dataset, $V_{a,i}$ and $V_{p,i}$ are the actual and predicted values, respectively, in the dataset. Table 1 displays the MSE and MAE results for the ANN models after 30 Epochs, where an Epoch represents the processing of all data once through several iterations, possibly involving data batches. The performance evaluation utilized five-fold cross-validation. Remarkably, all ANN models achieved an average MSE below 0.003, indicating an accurate prediction of the target variables. This successful outcome demonstrates the models’ ability to learn and capture the values of $\Phi$, *n*, and $R_s$, while effectively capturing the target variable trends. These findings underscore the impressive potential of ANN models to analyze and predict the internal parameters of an SPD, even without prior knowledge of the nonlinear thermionic emission (TE) expression for SBDs.

## Conclusions

ML models can be valuable tools that complement researchers, offering significant time savings in the device development cycle and adaptability to various fields. In this study, we successfully designed and developed diverse ANN-based models to analyze and extract the internal parameters of a Schottky photodiode (SPD). These models were trained and evaluated on 36 SPD datasets, yielding remarkable results. With an MSE and MAE score below 0.003, the ANN models accurately learned the internal parameters, predicting barrier height, series resistance, and ideality factor. It is worth noting that the ANN models also demonstrate their utility as a tool for post-publication validation of the results of earlier work that was based on the pedantic Cheung–Cheung method. Notably, the models demonstrated their ability to estimate unknown photodiode light responses under different illuminations and voltage settings without overfitting. This underscores their reliability. The utility of ML models to researchers lies in reducing the time-consuming repetition of experiments, enabling the generation of reliable internal parameters from prior data. By streamlining analysis tasks, researchers can now dedicate more attention to critical aspects of their investigations, thereby improving productivity.

## Data Availability

The datasets generated during and/or analyzed during the current study are available in the Google Colaboratory repository^26–28^, and figshare^25^.

## References

[1] Ping, H., Stoyanovich, J. & Howe, B. Datasynthesizer: Privacy-preserving synthetic datasets. In *Proceedings of the 29th International Conference on Scientific and Statistical Database Management*, 1–5. 10.1145/3085504.3091117 (2017).

[2] Gu Z (2023). Extracting accurate parameters of photovoltaic cell models via elite learning adaptive differential evolution. Energy Convers. Manag..

[3] Ruiz Euler H-C (2020). A deep-learning approach to realizing functionality in nanoelectronic devices. Nat. Nanotechnol..

[4] Robbins, H. & Monro, S. A stochastic approximation method. In *The Annals of Mathematical Statistics* 400–407 (1951).

[5] El-Mahalawy AM, El-Safty KH (2021). Classical and quantum regression analysis for the optoelectronic performance of NTCDA/p-Si UV photodiode. Optik.

[6] Chianese G, Franciosa P, Nolte J, Ceglarek D, Patalano S (2022). Characterization of photodiodes for detection of variations in part-to-part gap and weld penetration depth during remote laser welding of copper-to-steel battery tab connectors. J. Manuf. Sci. Eng..

[7] Lapointe S (2022). Photodiode-based machine learning for optimization of laser powder bed fusion parameters in complex geometries. Addit. Manuf..

[8] Chianese G, Franciosa P, Sun T, Ceglarek D, Patalano S (2022). Using photodiodes and supervised machine learning for automatic classification of weld defects in laser welding of thin foils copper-to-steel battery tabs. J. Laser Appl..

[9] Hammond WT, Mudrick JP, Xue J (2014). Balancing high gain and bandwidth in multilayer organic photodetectors with tailored carrier blocking layers. J. Appl. Phys..

[10] Hiramoto M, Miki A, Yoshida M, Yokoyama M (2002). Photocurrent multiplication in organic single crystals. Appl. Phys. Lett..

[11] Liang G, Cui T, Varahramyan K (2003). Electrical characteristics of diodes fabricated with organic semiconductors. Microelectron. Eng..

[12] Genty G (2021). Machine learning and applications in ultrafast photonics. Nat. Photonics.

[13] Sze SM, Li Y, Ng KK (2021). Physics of Semiconductor Devices.

[14] Nicollian EH, Brews JR (1982). MOS (Metal Oxide Semiconductor) Physics and Technology.

[15] Rhoderick, E. H. & Williams, R. H. *Metal-Semiconductor Contacts* (Clarendon Press, 1988).

[16] Durmuş H, Atav Ü (2011). Extraction of voltage-dependent series resistance from IV characteristics of Schottky diodes. Appl. Phys. Lett..

[17] Norde H (1979). A modified forward I-V plot for Schottky diodes with high series resistance. J. Appl. Phys..

[18] Cheung S, Cheung N (1986). Extraction of Schottky diode parameters from forward current-voltage characteristics. Appl. Phys. Lett..

[19] Gromov D, Pugachevich V (1994). Modified methods for the calculation of real Schottky-diode parameters. Appl. Phys. A.

[20] Ocaya R, Yakuphanoğlu F (2021). Ocaya–Yakuphanoğlu method for series resistance extraction and compensation of Schottky diode I-V characteristics. Measurement.

[21] Google Colaboratory. https://research.google.com/GoogleColaboratory/faq.html. Verified: 2023-04-22.

[22] Ocaya, R. *et al.* Graphene-oxide doped 2.9.16.23-tetrakis-4-$$\{$$4-[(2E)-3-(naphthalen-1-yl) prop-2-enoyl] phenoxy$$\}$$-phthalocyaninato cobalt (II)/Au photodiodes. *Synth. Metals* **209**, 164–172. 10.1016/j.synthmet.2015.07.016 (2015).

[23] Ocaya R (2017). Analysis of photoconductive mechanisms of organic-on-inorganic photodiodes. Phys. E Low Dimens. Syst. Nanostruct..

[24] Mekki A (2016). New photodiodes based graphene-organic semiconductor hybrid materials. Synth. Metals.

[25] Ocaya, R. O. *et al.* Dataset for Schottky photodiodes for machine language models. *figshare*10.6084/m9.figshare.22679278 (2023).

[26] Artificial Neural Network SBD Machine language model on Google Colaboratory. https://colab.research.google.com/drive/1iA6qP6CXS6X-mS0GW5fCmJi4i78v0rai?usp=sharing (accessed 22 Apr 2023).

[27] Linear Regression SBD Machine language model on Google Colaboratory. https://colab.research.google.com/drive/1nLPHjyX-TpOXnhXOls3A4N7D-rhdBmK1?usp=sharing (accessed 22 Apr 2023).

[28] Decision Tree SBD Machine language model on Google Colaboratory. https://colab.research.google.com/drive/1NAIl-xTAbijJB84SYVG3Uh16jybEon30?usp=sharing (accessed 22 Apr 2023).

[29] Pedregosa F (2011). Scikit-learn: Machine learning in Python. J. Mach. Learn. Res.

[30] Kingma, D. P. & Ba, J. Adam: A Method for Stochastic Optimization. 10.48550/arXiv.1412.6980 (2017).

